# Comparison of ventriculoperitoneal shunt to lumboperitoneal shunt in the treatment of posthemorrhagic hydrocephalus: A prospective, monocentric, non-randomized controlled trial

**DOI:** 10.1097/MD.0000000000020528

**Published:** 2020-07-02

**Authors:** Tong Sun, Chao You, Lu Ma, Yikai Yuan, Jingguo Yang, Meng Tian, Yicheng Zhou, Junwen Guan

**Affiliations:** a Department of Neurosurgery; b West China Brain Research Center; c Neurosurgery Research Laboratory, West China Hospital, Sichuan University, Chengdu, Sichuan, P.R. China.

**Keywords:** clinical outcomes, comparison, lumboperitoneal shunt (LPS), post-hemorrhagic hydrocephalus, ventriculoperitoneal shunt

## Abstract

**Background:**

Ventriculoperitoneal shunt (VPS) surgery remains the most widely accepted and used option method to treat post-hemorrhagic hydrocephalus (PHH) worldwide while lumboperitoneal shunt (LPS) serves as an effectively alternative treatment. However, the outcomes of VPS and LPS in the treatment of PHH have not been compared in a prospective trial.

**Methods and design:**

In this monocentric, assessor-blinded, non-randomized controlled trial, 75 eligible patients with PHH for each group will be recruited to compare the outcomes of VPS cohort with that of LPS cohort. Each participant is evaluated before surgery, at the time of discharge, 3, and 6 months after surgery by experienced and practiced assessors. The primary outcome is the rate of shunt failure 6 months after shunt surgery. The secondary measure of efficacy is National Institute of Health stroke scale, together along with Glasgow coma scale, modified Rankin Scale, and Evans index at the evaluation point. A favorable outcome is defined as shunt success with an improvement of more than 1 point in the National Institute of Health stroke scale. Complication events occurring within 6 months after surgery are investigated. A serious adverse events throughout the study are recorded regarding the safety of shunts.

**Discussion:**

The results of this trial will provide evidence for the treatment options for patients with PHH.

## 1 Introduction

Post-hemorrhagic hydrocephalus (PHH) is featured as the ventriculomegaly as a result of intraventricular hemorrhage (IVH), intraparenchymal hemorrhage, or subarachnoid hemorrhage (SAH) occurring secondary to traumatic brain injury (TBI) or hemorrhagic stroke.^[1–3]^ PHH could increase the intracranial pressure and damage periventricular white matter leading to neurological function defects, however, the pathogenesis remains controversial to date.^[1]^ Early studies suggested obstruction of intraventricular cerebrospinal fluid (CSF) flow induced by blood clot or fibrin was contributed to the occurrence of PHH while recent study indicated that inflammation-associated CSF hypersecretion of choroid plexus epithelium leaded to PHH.^[4]^ Despite of the debates, surgical intervention through CSF diversion remains the standard treatment for patients with PHH. CSF shunts, including ventriculoperitoneal shunt (VPS) and lumboperitoneal shunt (LPS), and endoscopic third ventriculostomy (ETV) remain the main treatment options, efficiently alleviating the symptoms immediately after surgery. VPS surgery is the most widely accepted and used option to treat PHH worldwide while LPS and ETV serve as effectively alternative treatments.^[5,6]^ However, the optimal treatment is still controversial.

With the emergence of neuro-endoscopic technologies in the1990s, a great deal of attention was given to the application of ETV for hydrocephalus, particularly non-communicating hydrocephalus.^[5]^ But the indications for performing ETV have recently broadened to communicating types of hydrocephalus.^[5]^ Based on the results of a randomized controlled trial, VPS is the superior method as a result of better neurological outcomes and lower incidence of severe complications while comparing to ETV.^[7]^ LPS, diverting the accumulated CSF from spinal subarachnoid space to peritoneal cavity, has some advantages over VPS, particularly the avoidance of brain injury, which promoted Japanese Neurosurgeons approval it as the first-line treatment for patients diagnosed as idiopathic normal pressure hydrocephalus (INPH).^[8–10]^ However, a prospective trial in Japan that analyzed the outcomes of patients with INPH treated by LPS and compared with a historical VPS control suggested the risk of shunt revisions of LPS was higher than that of VPS (LPS vs VPS: 7% vs 1%) at 1 year after surgery.^[8]^ To date, no completed prospective trials has compared VPS with LPS in the treatment of PHH.

## 2 Methods and design

### 2.1 Objective

In this trial, we will evaluate the efficacy and safety of CSF shunts in the treatment of PHH. The objective of this trial is to compare the outcomes of VPS cohort with that of LPS cohort.

### 2.2 Study design

The Shunting Outcomes of Post-hemorrhagic Hydrocephalus: Phase I (SOPH-1) is a monocentric, assessor-blinded, non-randomized controlled trial. 75 eligible participants for each group would be recruited. Neurosurgeons with extensive experience in the procedure conduct both types of shunt implantation. Each participant is evaluated before surgery, at the time of discharge, 3, and 6 months after surgery by experienced and practiced assessors. The primary outcome is the rate of shunt failure 6 months after shunt surgery. The secondary measure of efficacy is National Institute of Health stroke scale (NIHSS), together along with Glasgow coma scale (GCS), modified Rankin Scale (mRS), and Evans index at the evaluation point. A favorable outcome is defined as shunt success with an improvement of more than 1 point in the (NIHSS). Complication events occurring within 6 months after surgery are investigated. A serious adverse events (SAEs) throughout the study are recorded regarding the safety of shunts.

### 2.3 Recruitment and eligibility

**Figure 1:**
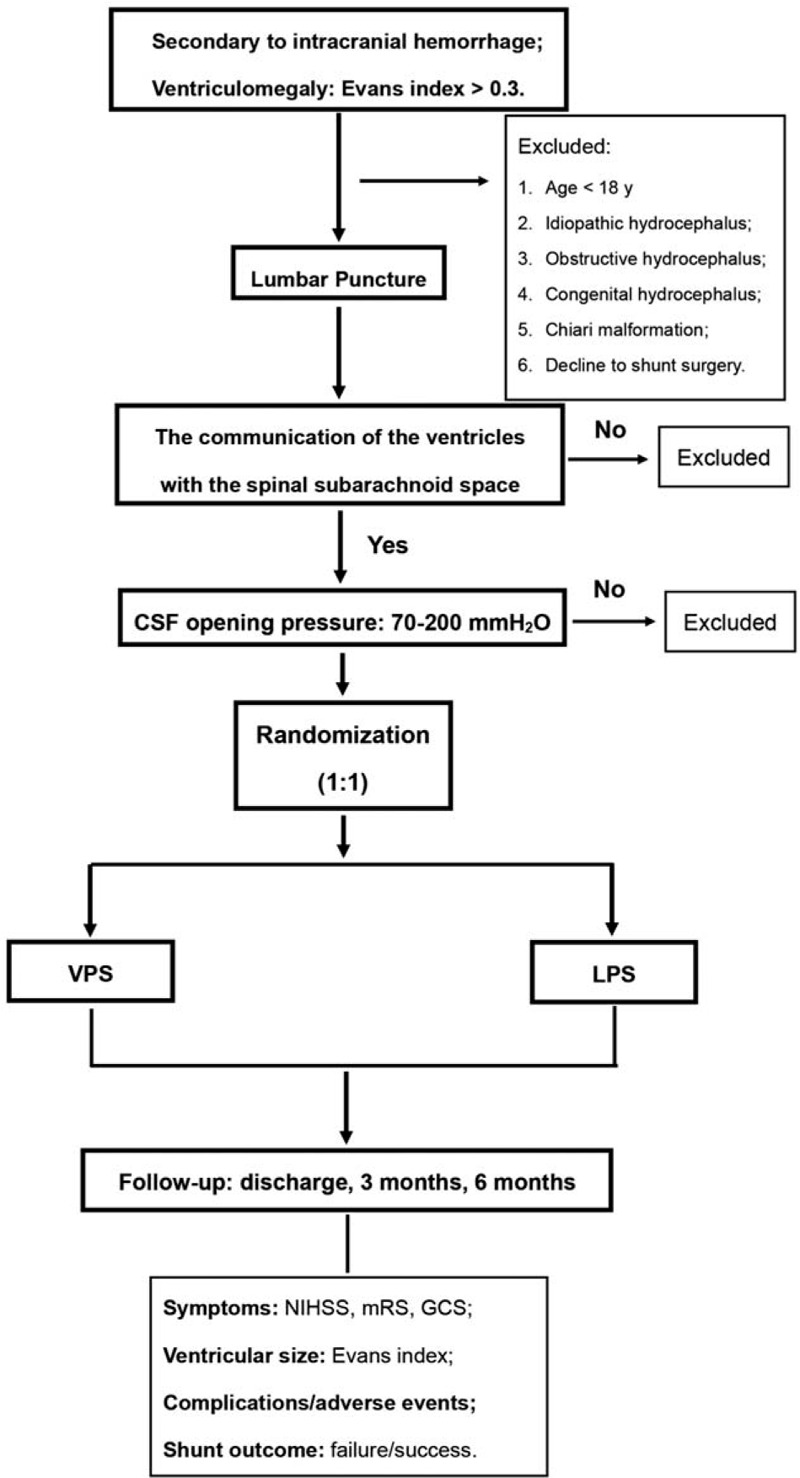
Flow chart of the selection of patients. CSF = cerebrospinal fluid, GCS = Glasgow coma scale, mRS = modified Rankin scale, LPS = lumboperitoneal shunt, NIHSS = National Institute of Health stroke scale, VPS = ventriculoperitoneal shunt.

Participants would be recruited on outpatient department of West China Hospital of Sichuan University since June 2020. As shown in Figure 1, once the participants are determined to be a potential candidate for shunt implantation, they would be admitted and performed a lumbar puncture in the lateral recumbent position to demonstrate the communication of the ventricles with the spinal subarachnoid space and test the CSF opening pressure. Each subject will receive financial compensation.

### 2.4 Inclusion criteria

1. Adult (Age >=18 years);
2. Evans index > 0.3;
3. Onset secondary to subarachnoid hemorrhage, intraventricular hemorrhage or intraparenchymal hemorrhage;
4. non-obstructive hydrocephalus;
5. The communication of the ventricles with the spinal subarachnoid space is evident through lumbar puncture and CSF opening pressure is 70-200 mmH_2_O.

### 2.5 Exclusion criteria

1. Idiopathic hydrocephalus;
2. Congenital hydrocephalus;
3. Obstructive hydrocephalus;
4. Chiari malformation;
5. CSF opening pressure is over 200 mm H_2_O or under 70 mm H_2_O;
6. Decline to shunt implantation.

### 2.6 Sample size

Previously published reports indicated that the rate of VPS failure is around 15.0% for adult patients with PHH, compared with a rate of 35.0% for LPS cohorts according to our previous study.^[11–14]^ We calculate that a sample of 70 will be required in this clinical trial with a significance level of 5% (2-sided) and a power of 80% to demonstrate a 20% difference in rate of shunt failure. Considering about the loss to follow-up, the sample size is enlarged to 75 for each group.

### 2.7 Randomizing and blinding

Given consent for participation and met eligibility, patients would be assigned groups. The assignment will be performed according to self-selection or administrator selection rather than through randomization and is kept secret by the statisticians who are independent of this study. It is not possible to blind participants to allocation, but data collection teams and analysts are blind to allocation and the intervention clinicians will not participate in any assessment.

### 2.8 Intervention

Neurosurgeons with extensive experience in the procedure conduct both types of shunt implantation and they will be trained centrally in advance and reach uniform standard. Shunt system is obtained from Medtronic, Inc, Minnesota, USA. Initial pressure for the shunt system is set to the highest level (2.5 level) before surgery.^[15]^ Shunt function is checked when there is no improvement in clinical symptoms or when tight high-convexity and medial subarachnoid spaces, enlarged sylvian fissures, or acute callosal angles were observed.^[16]^ The pressure setting is lowered by 1 step (0.5 level) with careful consideration of the patient's safety.

### 2.9 VPS

VPS implantation is conducted under general anesthesia and the patients are positioned in the supine position with the head turned to the left. Access to the lateral ventricle is obtained through scalp incision, skull drilling, and dura incision. Peritoneal access is obtained via a minimal invasive incision or split trocar access. A subcutaneous tunneler is passed from the abdominal incision to the cranial incision. The valve is placed at the cranial incision with a 3-point fixation to the subcutaneous tissue. Once the cranial catheter is connected to the valve and the valve is connected to the peritoneal tubing with confirmation of adequate CSF flow, the peritoneal catheter is inserted into the peritoneal space.

### 2.10 LPS

LPS implantation is conducted under general anesthesia and the patients are positioned in the lateral position. A lumbar catheter is inserted through the L3/4 or L2/3 interlaminar space into the spinal subarachnoid space. The catheter was then placed in a subcutaneous pocket made at the flank region. Peritoneal access is obtained via a minimal invasive incision or split trocar access. A tunneler is passed from the abdominal incision to the flank region, and then to the lumbar incision. The valve is placed at the frank region with a 3-point fixation to the subcutaneous tissue. Once the lumbar catheter is connected to the valve and the valve is connected to the peritoneal tubing with confirmation of adequate CSF flow, the peritoneal catheter is inserted into the peritoneal space.

### 2.11 Outcomes

As shown in Table 1, each participant is evaluated before surgery, at the time of discharge, and 3 and 6 months after surgery by experienced and practiced assessors other than the attending neurosurgeons, including neurologists, psychiatrists, clinical psychologists, and physical therapists.

**Table 1:**
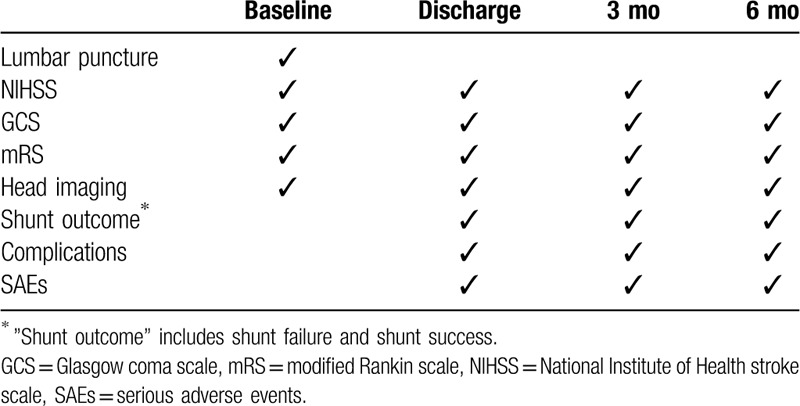
Study schedule.

### 2.12 Primary outcome

The primary outcome is the rate of shunt failure 6 months after shunt surgery. According to previous study, shunt failure is defined as the occurrence of clinical or radiological signs of shunt obstruction, breakage, tubing exposure, malfunction, or infection requiring shunt revision. Shunt success is defined as the absence of shunt failure.

### 2.13 Secondary outcomes

The secondary outcomes include NIHSS, CGS, mRS, and Evans index at the time of discharge, 3 and 6 months after shunt surgery. A favorable outcome is defined as shunt success with an improvement of more than 1 point in the NIHSS at the evaluation point. Evans index is tested thorough axial CT scan. Complication events throughout the study within 6 months after shunt implantation is investigated. Complication events occurring at any time within 6 months after cranioplasty are investigated.

SAEs occurring at any time is recorded to determine the safety, which is defined as death, an event that could lead to death, an event that require hospitalization or an extension of the hospitalization period, or disability, which is related to the treatment.

### 2.14 Data collection

The baseline characteristics and follow-up data will be collected by experienced and practiced assessors. The data collection form and the plan are shown in Table 1. Their family members will help them if they have difficulties in completing the evaluation. Any adverse events occurring during the study period are documented. Paper-based or electronic data will be recorded in the data collection form in time.

### 2.15 Data and safety monitoring

An independent data monitoring committee (DMC), including physicians, statisticians, and data analysts, will monitor the safety and efficacy of this trial and identify if there is a need to make adjustments. Members of the DMC will assess the trial once a month to review the study data.

### 2.16 Statistics analysis

All data are analyzed using the statistical software program SPSS version 19 (IBM, Armonk, NY). Probability values (*P*) less than .05 is considered to have statistical difference. Categorical variables are statistically descried as number (percent). Normal-distribution quantitative data are statistically described as arithmetic mean ± standard deviation (SD) and to compare the difference between the 2 groups, independent samples *t-*test is used. To compare the 2 groups on the data which followed non-normal distribution and are statistically described as median (range), Wilcoxon rank sum test is used. Chi-squared test is used to compare the 2 groups on categorical variables (Fisher's exact test is used while appropriate). Shunt-success rate curve is obtained using the method of Kaplan–Meier and log-rank test is used to compare the difference between the 2 groups.

### 2.17 Withdrawal and dropout

The researchers will record the reason for withdrawal and dropout if any participants choose dropout, or it is not appropriate to continuously participate in this trial.

## 3 Discussion

With the advent of silastic tubing and programmable pressure valve, CSF shunts diverting the accumulated CSF to the peritoneal cavity remained the standard treatment.^[17,18]^ The most common used type of shunt was VPS while LPS emerged as an effective alternative treatment in 1950s.^[19]^ While an unacceptably higher incidence of shunt failure in the treatment of INPH was observed, few studies tested the benefits of LPS surgery in the treatment of PHH. A retrospective study recently indicated patients with PHH treated by LPS and VPS obtained equal clinical outcomes.^[2]^ However, the best treatment still remains controversial.^[20]^ In this regard, we conducted a prospective, randomized controlled trial to compare the safety and efficacy of VPS cohort with that of LPS cohort with PHH. The primary outcome is the rate of shunt failure 6 months after shunt surgery. The secondary measure of efficacy is the improvement of symptoms and ventricular size, and the incidence of complications at the evaluation point within 6 months. To better of our knowledge, the current study is the first randomized controlled trial that compares the long-term outcomes of VPS with that of LPS in the treatment of PHH. The results of this trial will provide evidence for the optimal treatment options for patients with PHH and may generate the discussion about the optimal treatment. Despite of the strengths, there are still some questions that need to be discussed.

First, the indication and contraindication of LPS implantation remains controversial since the lack of broad consensus and standardized criteria.^[10,11]^ Diverting the accumulated CSF from spinal subarachnoid space to peritoneal cavity, LPS is only suitable and effective for non-obstructive hydrocephalus. In this light, participants those who are suspected for obstructive hydrocephalus based on brain imaging would be excluded from this trial.

On the other hand, it is generally accepted that low-pressure, negative-pressure, and high-pressure hydrocephalus, are not suitable for the LPS implantation. However, the acceptance is not evidence-based. Normal-pressure hydrocephalus is defined as the CSF opening pressure in the range of 70 to 245 mm H_2_O measured by lumbar puncture in the lateral recumbent position according to the Western INPH guideline, comparing to a CSF opening pressure under 200 mm H_2_O based on Japanese INPH guideline.^[21,22]^ Taken together, a range of 70 to 200 mm H_2_O is considered in this study since pressures that are dramatically higher or lower than this range are not suitable for the upcoming LPS implantation.

The current study, however, still has some limitations. First, it is single-center and non-randomized study. Second, medical conditions and surgeons’ experiences are contributed to the postoperative outcomes. In this regard, personnel will be trained centrally in advance and reach uniform standard.

## 4 Ethic and dissemination

The trial is in compliance with the Guidelines for Good Clinical Practice and the Declaration of Helsinki (2002) of the World Medical Association. This study protocol was prepared according to the Standard Protocol Items: Recommendations for Intervention Trials (SPIRIT Checklist). The study is registered prior to data collection through Chinese Clinical Trial Registry in January 2020 (ChiCTR2000028766). The Institutional Review Board of West China Hospital had approved the current study. All patients will be fully informed the potential treatments, potential complications following shunt surgery, responsibilities during the trial, and they will sign the informed consent before joining in this trial. The results will be published in peer-reviewed journals and at national and international conferences.

## 5 Patient and public involvement

No patient or public is involved in the design, recruitment, or conduct of this research.

## Author contributions

TS is responsible for providing critical review of concept, study design, and protocol writing. JG and CY are responsible for conceiving the idea for the present study, study design, obtaining funding, and protocol writing. MT, LM, and YZ are involved in trial design, supervision, and revised the protocol. YY ang JY are responsible for statistical analysis, investigation, and data curation. All authors approve the final manuscript.

**Conceptualization:** Junwen Guan, Chao You, Tong Sun.

**Data curation:** Yikai Yuan, Jingguo Yang.

**Formal analysis:** Yikai Yuan, Jingguo Yang.

**Funding acquisition:** Junwen Guan, Chao You.

**Investigation:** Yikai Yuan, Jingguo Yang.

**Methodology:** Tong Sun, Junwen Guan, Chao You, Meng Tian, Lu Ma, Yicheng Zhou.

**Project administration:** Chao You.

**Software:** Tong Sun.

**Supervision:** Junwen Guan, Chao You.

**Validation:** Junwen Guan, Chao You.

**Writing – original draft:** Tong Sun.

**Writing – review & editing:** Junwen Guan, Chao You, Lu Ma, Meng Tian, Yicheng Zhou.
